# Traitement chirurgical des Affections inflammatoires et néoplasiques de l'Utérus et de ses Annexes

**Published:** 1894-03

**Authors:** 


*Traitement chirurgical des Affections inflammatoires et néoplasiques de l'Utérus et de ses Annexes.* Par E. DOYEN. Pp. 128. Paris: BUREAUX DES *Archives provinciales de Chirurgie.* 1893.

This work, which is based upon 324 operations on the uterus and its appendages, has been revised and enlarged since its original appearance in the *Archives provinciales de Chirurgie* in 1892.

The author has for some time past made a point of examining the exudate in cases of pelvic inflammatory mischief, and finds that the most frequent organisms are the streptococcus and the gonococcus.

The work is mainly divided into two portions—the first treating of inflammatory affections, and the second of new growths. For inflammatory mischief, the author strongly urges vaginal hysterectomy as the operation best suited to most circumstances, although he admits that in certain well-localised perimetric inflammations a simple incision in the inguinal region (sub-peritoneal) would meet the case; and that laparotomy is very good when the affection is unilateral and without many adhesions, and for painful retroversion without metritis or severe lesions of the appendages.

So firm is the author's belief in the superiority of vaginal hysterectomy for these inflammatory lesions, that he almost disarms criticism by stating, like Segond, that the opponents of this method are "*ceux qui la connaissent le moins.*" Had it not been for this statement, we might have been bold enough to have expressed our conviction that the treatment which he advocates is not always so desirable or necessary when other less dangerous procedures would fulfil the indications for operation.

In the second part of the book, which is devoted to new growths, the author describes his operations for abdominal and vaginal hysterectomy. These are novel in many points and well worth consideration. Their description is accompanied by figures and diagrams, which elucidate the text and enhance the value of the work.

